# HIV self-testing implementation, distribution and use among female sex workers in Cotonou, Benin: a qualitative evaluation of acceptability and feasibility

**DOI:** 10.1186/s12889-022-12917-3

**Published:** 2022-03-26

**Authors:** Marianne Boisvert Moreau, Frédéric D. Kintin, Septime Atchekpe, Georges Batona, Luc Béhanzin, Fernand A. Guédou, Marie-Pierre Gagnon, Michel Alary

**Affiliations:** 1 Centre de recherche du CHU de Québec – Université Laval, Québec, QC Canada; 2 Département de médecine sociale et préventive, Université Laval, Québec, QC Canada; 3 Dispensaire IST, Centre communal de santé de Cotonou-Zone1, Cotonou, Bénin; 4 Organisation pour la promotion de la santé et le développement communautaire, Cotonou, Bénin; 5 École doctorale pluridisciplinaire “Espace cultures et développement”, Filière sociologie du développement, Université d’Abomey-Calavi, Godomey, Bénin; 6 World Health Organization, Brazzaville, Congo; 7 École nationale de formation des techniciens supérieurs en santé publique et en surveillance épidémiologique, Université de Parakou, Parakou, Bénin; 8 VITAM – Centre de recherche en santé durable, Québec, QC Canada; 9 Faculté des sciences infirmières, Université Laval, Québec, QC Canada; 10 Institut national de santé publique du Québec, Québec, QC Canada

**Keywords:** HIV, HIV self-testing, Female sex workers, Acceptability, Feasibility, Theoretical Domains Framework, Benin

## Abstract

**Background:**

In Benin, the burden of HIV is disproportionately high among female sex workers (FSWs). HIV testing and knowledge of status are starting points for HIV treatment and prevention interventions. Despite the importance given to testing services in HIV control, its uptake among FSWs remains suboptimal in Benin. HIV self-testing (HIVST) may be useful for increasing testing rates in FSWs.

**Methods:**

We conducted a pilot study of the distribution of saliva-based HIVST among FSWs in Cotonou and its surroundings, Benin. The HIVST promotion and distribution model included three complementary strategies: community-based, facility-based and secondary distribution. In this qualitative study, we explored the elements influencing HIVST implementation, distribution and use among FSWs. We assessed HIVST acceptability and feasibility in this population. We conducted 29 semi-structured individual interviews with FSWs. Data were interpreted with a thematic analysis method, using the Theoretical Domains Framework.

**Results:**

Only two FSWs (6.9%) were aware of HIVST before participating in the study. All participants were interested in using HIVST if available in Benin. Many advantages of HIVST were mentioned, including: autonomy, privacy, accessibility, time saving, and the fact that it is a painless test. Barriers to the use of HIVST included: the fear of unreliability, the lack of psychological support and medical follow-up and the possibility of result dissimulation. Participants thought HIVST was easy to use without assistance. HIVST enabled linkage to care for a few FSWs in denial of their HIV-positive status. No case of suicide or violence associated with HIVST was reported. HIVST secondary distribution within FSWs social network was well received. FSWs’ boyfriends and clients showed interest in using the device. Some FSWs reported using HIVST to practice serosorting or to guide their decisions regarding condom use.

**Conclusions:**

Our study shows a very high level of acceptability for HIVST among FSWs in Cotonou and its surroundings. Results also demonstrate the feasibility of implementing HIVST distribution in Benin. HIVST should be implemented in Benin quickly and free of charge for all individuals at risk of HIV. HIVST offer should be integrated with comprehensive sexual health and prevention services.

**Supplementary Information:**

The online version contains supplementary material available at 10.1186/s12889-022-12917-3.

## Background

Despite progress in the last decades, sub-Saharan Africa remains the region of the world that carries the highest human immunodeficiency virus (HIV) burden. In this region, sex work is an important driver of the epidemic [1] and female sex workers (FSWs) have a heavy burden of HIV, with a prevalence estimated to be at least 12 times higher than for women of the general population [2]. In West and Central Africa, in 2019, 4.9 million people were living with HIV, 240,000 were newly infected and 140,000 died of conditions related to the acquired immunodeficiency syndrome (AIDS) [3]. The Joint United Nations Programme on HIV/AIDS (UNAIDS) data indicate that among all people living with HIV in West and Central Africa at the end of 2019, only 68% knew their serological status [3].

Benin is not spared by the HIV epidemic. In this country, 75,000 people were living with HIV and 3,500 were newly infected in 2019 [4]. As in most West African countries, the HIV epidemic is concentrated among key populations in Benin. Beninese FSWs have a disproportionate burden of HIV, with an estimated prevalence of 8.5% in 2019, between eight and nine times higher than for the general population, among which the prevalence was 1.0% the same year [4]. In Benin, transactional sex is the driving force of the HIV epidemic [5]. FSWs represent the main core group in the local HIV transmission dynamics, and their clients act as a bridging population, transmitting the infection from the core group of FSWs to their sexual partners [5]. Beninese FSWs are particularly vulnerable to HIV acquisition and transmission due to the nature of the work, its practice in precarious conditions, the obstacles to condom use and the inequitable access to appropriate health services [6]. FSWs generally have little control over these factors, due to social marginalization and sex work criminalization [6].

HIV testing is the first step of the HIV care cascade and the entry point to HIV treatment and support services. According to the most recent UNAIDS targets, by 2025, within all sub-populations and age groups, 95% of all people living with HIV should know their status, 95% of those diagnosed should receive antiretroviral treatment, and 95% of those on treatment should achieve viral suppression [7]. However, in Benin, FSWs knowledge of their serological status remains insufficient. In a 2017 national surveillance survey, the percentage of FSWs who had been tested for HIV in the past 12 months was 59.2% in Benin and 66.1% in Cotonou [8]. Qualitative and quantitative studies carried out on the issue of HIV testing among FSWs in Benin reveal the existence of personal factors that hinder their use of testing in formal health centers [9–11]. Fear or lack of motivation to go to a health center, perception of a lack of quality of care offered there, stigma, discrimination and fear of breach of confidentiality are cited among these factors [10, 11]. There are also various structural obstacles related to access to health care, such as the lack of knowledge of free services offered in HIV testing centers, the remoteness of the centers and the high cost of transportation [11].

The HIV self-test is a rapid diagnostic orientation test used by a person who wants to know his or her status. HIV self-testing (HIVST) is a process by which the user takes a sample (oral fluid or blood), performs the test and interprets the result alone, often in a private setting [12]. To enable more people living with HIV to be diagnosed, the World Health Organization (WHO) recommends HIVST as a complementary approach to standard testing services [12]. Much evidence show that HIVST is associated with an increased uptake and frequency of HIV testing, which leads to an increased number of people aware of their HIV status [13–17]. Therefore, the introduction of HIVST in Benin may help remove some of the barriers faced by key populations and improve their access to HIV testing.

Since the HIV response is one of Benin government’s priorities, the *Programme santé de lutte contre le Sida* adopted in 2017 a strategic framework for demedicalization of HIV testing, which proposes HIVST integration into the new testing guidelines. The HIVST promotion planned in the national strategy, which is not yet effective, would shift the service offer to community settings, particularly among hard-to-reach populations who have limited access to existing health services. Self-tests distribution in Cotonou and its surroundings could improve the access and use of HIV testing services among high-risk populations, such as FSWs.

Accordingly, information on the determinants of HIVST acceptability and feasibility is needed in countries like Benin that are considering its national implementation. To our knowledge, no data about distribution strategies nor about HIVST feasibility among the FSW population in Benin was available before the beginning of our study. To fill this gap, this study explored different factors that may facilitate or limit HIVST implementation, distribution and use among FSWs through a descriptive qualitative methodology. The study aimed at widening current knowledge by using the Theoretical Domain Framework (TDF) to inform the development of a tailored HIVST program in Benin.

## Methods

### Study design and population

The focus of this paper is the qualitative component of a larger mixed-methods study conducted in 2020–2021 in Cotonou, the economic capital and largest city of Benin, and its largest suburb, Abomey-Calavi. The qualitative study was organized into 29 individual in-depth interviews with FSWs. Women identifying themselves as professional FSWs (e.g. earning most of their revenue from sex work) and recently frequenting identified sex work sites in Cotonou and its surroundings that were operational during the course of the study constituted the study population. Women were eligible to participate if they were born female, aged 18 or older, declared that sex work was their main source of income, received and performed saliva-based HIVST (OraQuick ® HIV Self-Test, OraSure Technologies, Bethlehem, PA, USA) in the context of this study, were able to understand the information given about the study and provided informed consent.

### Program setting

The HIVST promotion and distribution model included three complementary strategies that were intended to encourage broad coverage while offering FSWs the choice of the preferred delivery channel. They were: community-based, facility-based and secondary distribution. Two HIVST promotion and distribution episodes were conducted, from June 22^nd^ to July 20^th^, 2020 and from November 9^th^ to December 7^th^, 2020.

Four non-governmental organizations (NGOs) from Cotonou and Abomey-Calavi that already had an anchor in Beninese sex work environments were responsible for the community-based promotion and distribution through field activities. Community-based distribution in sex work sites and FSWs’ places of residence relied on eight community animators and 16 peer educators (PE) who worked under their supervision. Community animators and PEs teamwork in preventive interventions is a dominant mobilization approach that is known to be effective in the Benin sex work environment [18].

The facility-based distribution was carried out by two user-friendly health centers already dedicated to FSWs’ medical follow-up. In each facility, three trained agents were designated responsible of the HIVST promotion and distribution. FSWs who went to these facilities were given the possibility to self-test for HIV while they were waiting for their appointment or when they were seen by the doctor. To do so, a private space was specially set up in each clinic. FSWs also had the opportunity to use the self-test with the assistance of a trained officer, which gave them the opportunity to immediately undergo a confirmatory test in case of a reactive result. FSWs could also receive self-test kits to take home for themselves, their partners or other people of their social network. The two participating facilities provided medical follow-up, care, support and antiretroviral therapy (ART) for women found to be HIV-positive.

Secondary distribution gave FSWs the opportunity to redistribute HIV self-test kits to one or many contacts of their social networks, such as sexual partners, clients, friends or acquaintances. To do so, every FSW was offered three self-test kits per distribution episode. As part of the secondary distribution, FSWs were made aware of the importance to provide support to the people with whom they shared HIV self-test kits. FSWs were therefore asked to teach secondary beneficiaries how to properly use the device and were encouraged to refer and accompany them to a health care facility to confirm the diagnosis in case of a reactive self-test. Secondary beneficiaries were neither mandated to self-test in the presence of the FSW nor required to disclose their result to her.

### Recruitment and sampling

Participants were first identified by community animators or PEs, who approached FSWs for a preliminary agreement to voluntarily participate in a qualitative interview. The ones who demonstrated enthusiasm for the project were then put in touch with the research assistant, who ensured applicants' compliance with the eligibility criteria, provided them full information about their participation in the study and confirmed their agreement. Diversification criteria were used in the selection of participants in order to get the greatest diversity of attitudes towards HIVST. Thus, women who reported having experienced significant and unique situations (violence, having shared kits with different types of partners, refusal or reluctance of secondary beneficiaries to use self-tests) were more likely to be recruited to participate in an interview.

### Conceptual framework

The Theoretical Domains Framework (TDF) [19] was used to generate the interview guide (see Supplementary Material) and to carry out the thematic analysis of the data. The TDF is a synthesis of 33 different behavioral theories from which 128 constructs were identified. From these 128 constructs, only the most relevant to health behavior change were kept. In the latest version of the TDF, 14 domains are identified [20, 21]. This framework helps understand behavior change by exploring their determinants and provides useful information to design interventions. It was used in this study to explore health behaviors related to HIVST.

In this study, eight domains were explored during in-depth individual interviews: knowledge, reinforcement, social influences, intentions, beliefs about capabilities, behavioral regulation, beliefs about consequences and emotions. Knowledge explores participants awareness of HIVST and other screening methods. The reinforcement domain refers to all the factors that can either increase or decrease participants’ motivation or interest to use HIV self-tests. The domain of social influences explores all interpersonal processes that can cause individuals to change their thoughts, feelings or behaviors. The intention domain explores the participants' intention to use HIVST on a regular basis if made available at all times. Beliefs about capabilities are related to participants’ self-confidence about their capacity to use self-test kits as recommended. The behavioral regulation domain was used to evaluate participants anticipated sexual behavior changes with HIVST. The domain of beliefs about consequences explores the fears or apprehensions that FSWs might have in relation to the expected results of the HIV self-test. Finally, the domain of emotions was used to identify fear, anxiety or stress reactions related to the use or distribution of HIV self-tests. Two domains were not selected because they were less relevant to the objectives of this study (goals, and memory, attention and decision processes) and four were excluded because they were mainly related to elements already covered in one of the eight chosen domains (skills, social/professional role and identitiy, environmental context and resources, and optimism).

### Data collection

All 29 individual in-depth interviews were conducted by a qualitative research professional from the field team. These interviews were semi-structured using an interview guide based on the TDF as described above. The interview guide was developed and validated with the field team. Interviews were conducted in the language chosen by each participant (French, English or Fon) and were recorded with a digital device. Interviews lasted between 19 and 73 min, with a mean duration of 41 min.

### Data analysis

The interviews were transcribed verbatim. For a few participants who used local languages to express themselves, the excerpts were first translated into French. After verification by two researchers, digital transcriptions were imported in N’Vivo 1.3.1, a software that allows to manage and analyse qualitative data. Data were codified and analysed through a thematic content analysis that is appropriate to identify key elements, contradictions and recurrences between participants [22]. Deductive codes were developed based on the individual interview guide, and emergent themes were identified during subsequent transcript readings. The first codification was carried out by the first author (MBM) and then reviewed by one of the co-authors (MPG) with extensive experience in qualitative research. Codifications or interpretation discrepancies were discussed by the research team to reach a consensus.

### Ethical considerations

The study was approved by the National Health Research Ethics Committee of Benin and by the Research Ethics Committee of CHU de Québec – Université Laval. All participants provided informed written consent after receiving detailed information on the study. Actions were taken to ensure confidentiality and anonymity. At the end of the interview, each participant received 2,500 FCFA (approximately, 4.50$ US) to compensate for the time given to the study.

## Results

### Socio-demographic and behavioral characteristics of participants

A total of 29 FSWs were included in the analysis. Study participants sociodemographic and behavioral characteristics are provided in Table 1. The mean age of participants was 34.3 years. The majority of FSWs were divorced (55.2%) or single (34.5%). While most FSWs had a boyfriend (55.2%), none was cohabiting with a regular sexual partner. More than a quarter of the participants (27.6%) had no education. FSWs were mostly from Benin (37.9%), Nigeria (31.0%) and Togo (13.8%). Participants had an average of 3.8 years in sex work. Twenty-five FSWs (86.2%) were biological mothers. Five participants (17.2%) self-reported as seropositive after using the HIV self-test.

**Table 1:** Participants sociodemographic and behavioral characteristics (*N* = 29)

	**n (%)**
**Age in years** [mean ± SD]	**34.3** ± **7.6**
20—29	9 (31.0)
30—39	14 (48.3)
40—49	4 (13.8)
≥ 50	2 (6.9)
**Marital status**	
Divorced	16 (55.2)
Single	10 (34.5)
Widowed	3 (10.3)
Married	0 (0)
**In a relationship with a regular partner (husband or boyfriend)**	
Yes	16 (55.2)
No	13 (44.8)
**Cohabiting with a regular partner**	
Yes	0 (0)
No	29 (100)
**Highest education level achieved**	
No education	8 (27.6)
Primary school	6 (20.7)
Secondary school	13 (44.8)
Higher than secondary school	2 (6.9)
**Country of origin**	
Benin	11 (37.9)
Nigeria	9 (31.0)
Togo	4 (13.8)
Ghana	3 (10.3)
Other^1^	2 (6.9)
**Religion**	
Christianism	21 (72.4)
Islamism	6 (20.7)
No religion	2 (7.0)
**Number of years in sex work [mean ± SD]**	**3.8 ± 3.8**
< 2 years	11 (37.9)
2 – 3 years	5 (17.2)
4 – 5 years	9 (31.0)
> 5 years	3 (10.3)
Unknown	1 (3.4)
**Other source of income**	
None	13 (44.8)
Clothes or shoes sale	6 (20.7)
Hairdresser	3 (10.3)
Other^2^	7 (24.1)
**Number of children [mean ± SD]**	**2.4 ± 1.9**
0	4 (13.8)
1 – 2	13 (44.8)
3 – 4	8 (27.6)
≥ 5	4 (13.8)
**Number of dependants [mean ± SD]**	**5.6 ± 3.8**
1 – 2	5 (17.2)
3 – 4	8 (27.6)
5 – 6	6 (20.7)
≥ 7	10 (34.5)
**Self-reported HIV status**	
Positive	5 (17.2)
Negative	23 (79.3)
Unknown	1 (3.4)

*SD Standard deviation*

^*1*^*Cameroun, Burkina Faso*

^*2*^*Sewing, agriculture, bar service, aesthetics, food sale, charcoal sale*

### Knowledge

Participants’ knowledge about HIV conventional testing recommendations, HIVST and HIV prevention methods were explored. The majority of participants (62.1%) reported being tested for HIV regularly in formal health centers, at least every three months, while 27.5% said they were tested irregularly. Three FSWs had never been tested for HIV prior to their use of HIVST in this study.

The majority of participants were not aware of HIVST before their participation in this study. Out of the 29 participants, only two (6.9%) had heard of HIVST. While the first had known about it from another FSW, the second had had the opportunity to experience HIVST when she was a sexual health community animator in her country of origin, Togo.

Most participants stated that they used condoms during sexual intercourse, but some admitted that they do not use it every time. In general, FSWs seemed to give more importance to condom use with their clients than with partners they considered closer, as their boyfriends and their regular clients:

*“ As a client I asked him to wear the condom. It was once he became my boyfriend that we stopped using condoms when we had sex. "* (Participant #26).

### Reinforcement

#### Advantages and incentives

Many incentives about HIVST were mentioned by participants. The most frequently stated advantages of HIVST were its autonomy (51.7%) and privacy (37.9%), followed by the fact that it is a painless test (34.5%). Nine participants (31.0%) also liked HIVST for its accessibility. Table 2 summarizes the main incentives and advantages that were mentioned with verbatim examples to illustrate participants’ answers.

**Table 2:** Summary of incentives and advantages of HIVST reported by 29 FSWs

Autonomy	*" We are also independent with the self-test which can be done alone, at home, unlike conventional screening where you have to come to the health center and be dependent on the health worker. "* (Participant #20)
Privacy	*" You don't need to talk, or the people next door don't need to know what you're doing. And, whatever is your result, you get up, you go to your doctor who takes care of you. It’s good and discreet. "* (Participant #1)
Painless	*" I prefer the self-test to the conventional screening where you have your fingertip pierced. Because when my finger is pierced, I feel too much pain. "* (Participant # 9)
Accessibility	*“ With the self-test kit, we can check our HIV status ourselves at home at times, without necessarily having to go to the hospital. […] Because going to the medical center costs transportation money and is a waste of time for me. "* (Participant # 7)

#### Consequences and barriers

Although most of the participants had no apprehension about HIVST, some disincentives were mentioned during the interviews. Six women (20.7%) feared that the device may be unreliable because it uses a saliva sample rather than blood, which they had never heard of before. Six FSWs (20.7%) also talked about the possibility for users who test positive to conceal their results. Finally, three participants (10.3%) were worried that users would suffer from a lack of psychological support or medical follow-up after performing HIVST. Table 3 summarizes the main consequences, barriers and disincentives that were mentioned with verbatim examples to illustrate participants’ answers.

**Table 3:** Summary of consequences, barriers and disincentives of HIVST reported by 29 FSWs

Fear of unreliability	*“ I trust the standard test result more than the self-test result. Because for the standard test, you look for the virus directly in your blood, and we've known about it ever since. But the self-test is saliva. "* (Participant #10)
Possibility to conceal result	*“ The downside I find with the self-test is that there can be people who hide their positive HIV status and cut ties with others so they don't have to go to the hospital. "* (Participant #16)
Lack of psychological support	*" With [the self-test], if I'm all alone in my room and see a bad result, what would my reaction be? I can have a fit or I can be uncomfortable with the fear. But if someone else gives you [the test], they'll know how to calm you down first. […] If someone else is there, it is more reassuring to follow him. The person will try to calm you down, to relieve you with certain words, so that you come back to yourself. But if it's you alone, what would it be? I do not know. "* (Participant #25)
Lack of medical follow-up	*" A lot of people have taken the kits and will come and tell you that they have used it, but in reality, they have not used it. Maybe there are even some who threw it away. How will the doctor who's coming here know that someone has used it? He can't, since the girls are alone and didn't already like going for a test. "* (Participant #22)

Social harms associated with HIVST could also constitute barriers as they may decrease participants' interest in using it. Among the 27 participants asked about social harms related to HIVST, none had experienced or heard of any cases of suicide or violence. Two participants mentioned that they had left their boyfriends because of their refusal to use the HIV self-tests they had shared with them. However, these breakups did not appear to be directly related to HIVST since the two FSWs specified having lost confidence in their boyfriends, who dated several women without using protection and did not show interest in maintaining their own health.

### Social influences

#### Stigmatization

All participants agreed that stigmatization towards FSWs was real in Cotonou and in Benin. Most reported that the situation was very challenging for them. The stigmatization seems to be even worse for FSWs living with HIV. It was the case of one of the participants, who had been forced to move out from the sex work site where she worked and abandon ART a few months after obtaining her diagnosis, because of the rumors spreading among her peers:

*“ It's because everyone at the site where I work knows my HIV status. […] When I'm in my room, I hear people outside saying, ‘ It's because she's taking HIV medication that she's locked up and tucked away in her room all the time.’ It was because of what people know and said about me that I have decided to leave this site. "* (Participant #13)

*“ When I came back to Benin, I avoided going to take the drugs because I feared that my comrades would know that I was really taking ART. "* (Participant #13)

To find out if participants experienced situations where they felt stigmatized in connection with HIVST, they were asked if they feared being seen in possession of an HIV self-test or purchasing one in a pharmacy. The majority of FSWs were not afraid of being seen in possession of the device:

*“ Even if someone sees the kit in my hands, the person won't know what it's for. It's not just night girls who are at risk of infection and have the self-test kits! "* (Participant #19)

On the other hand, three participants mentioned that they would feel some discomfort if they were seen with the device:

*“ It's not a kit that you need to have on hand and show people, or have it and let people know for example. Having the HIV self-test on you could tell others that you work in prostitution. However, this work does not honor the one who practices it; we all know it's something bad. The day I was given the kits, I put them in the bottom of my bag. Frankly. "* (Participant #26)

#### Pride in being role models

Several FSWs said they were proud to be role models for others, by sharing HIV self-tests, teaching how to use the device and directly assisting secondary beneficiaries:

*" I appreciate the fact that through me two other people have been screened at the same time as me and we know our results […]. If this can be the case in the country, everyone will know their HIV status and understand how to take care of themselves. "* (Participant #8)

*" […] When I did [the self-test to him], he [the client] was happy, he thanked me, he told me that I was nice […]. I had never heard someone thank me so much. He told me that I care about his health apart from the money I'm looking for, that means I consider him, that I want him to be healthy. […] It was the way he liked it that made me do [the self-test] on others. "* (Participant #25).

### Beliefs about capabilities

#### Ease of use

FSWs’ personal confidence to use HIVST properly was explored. To do so, women were asked by which steps they had used the device. All participants said they found it easy to use the device on their own, and believed they could perform it without assistance:

*“ With the explanations received, no step was difficult for me to do the self-test. It is not a test that requires physical effort to exert. "* (Participant #3)

*“ When the self-test is well explained, no help is needed. "* (Participant #8)

Most participants (96.6%) were confident enough in their abilities to report, after using HIVST, that they knew their HIV status based on their interpretation of the result.

Still, a few FSWs experienced difficulties using the device. Five FSWs did not seem to know or remember how long they had to wait before reading the test result. Three participants said they had trouble opening the tube without spilling its content. Two women mentioned making a mistake or finding it hard to pass the spatula over their gums. Finally, a participant said she had rubbed the spatula on her tongue, which was not recommended.

#### Demonstrations, manufacturer's instructions and advices’ contribution

Most participants stated that the in-person demonstrations of the use of the device, the manufacturer's illustrated instructions and the community animators and PEs’ advices on how to interpret the results had increased their self-confidence in their ability to use the device properly.

*“ They demonstrated the use of the kit three times during the session, they explained and asked if it was clear to us. They gave us time to ask questions. It was clear to us. "* (Participant #20)

*“ On the kit’s instructions that they also showed, there were pictures and arrows that indicated how to do it and the sequence of steps from beginning to end. This was what I followed with my boyfriend to complete the self-test step by step until the end. "* (Participant #30)

### Intentions

Intention to use HIVST was very high in our study: all 29 participants said that they had the intention to use the HIV self-test if made available in Benin. We also wanted to know which distribution strategy participants prefered to receive HIV self-tests. The majority of participants wanted to receive the kits from NGOs dedicated to FSWs or health care facilities that they already visit on a regular basis:

*" […] I think we could leave the self-test kits with the NGO animators who distribute the condoms to us. So if we need them, we can resort to them. "* (Participant #7)

*“ […] Since we do our medical visits here to the medical center, I think we can put the kits there so we have access. "* (Participant #2)

Several women emphasized the importance for the devices to remain free of charge to maintain their attractiveness to FSWs:

*" It's possible in pharmacies, but there it would surely be sold? If it was, we wouldn't be able to have access; we don't have the means to deal with it. We should continue to give it to us for free. "* (Participant #21)

Moreover, many FSWs had the intention to share HIV self-tests within their social network if secondary distribution continued to be encouraged:

*“ If I'm given ten [HIV self-tests], I'll take. […] I am going to distribute it to my parents, my relatives, because it is very easy and very effective. "* (Participant #1)

### Behavioural regulation

A frequent concern with HIVST implementation is the possibility of risk compensation. It is feared that partners who get non-reactive results, feeling protected, may take more risks in their sexual practices. In our study, most participants (58.6%) affirmed that HIVST would have no influence on their sexual behaviours: they believed they would not use less the condom if they obtained non-reactive results with the HIV self-test. Still, many FSWs tended to cease condom use with their boyfriend when they both obtained non-reactive self-test results:

*“ I used to have sex with my boyfriend with condoms. But when I went to his house and he showed me his negative test result, it reassured me and I started having sex without condom with him. "* (Participant #10)

Some regular clients who had used HIVST as part of the secondary distribution and had obtained non-reactive results tried to convince FSWs to have unprotected sex with them:

*" There were even some [clients] who, after the negative result of their self-test, said to me: 'Now that you know my serological status and that you know that I do not have HIV, can we have sex without condom?' And I tell them I'm not going to have sex with them without a condom, because there's not just HIV out there. "* (Participant #25)

Similarly, a few FSWs mentioned that their boyfriends had used their non-reactive HIV self-test results as a negociation leverage to have unprotected sex:

*“ […] Since we got negative results from the self-tests, my boyfriend insists that we no longer use condoms in our intercourse. This is the source of our arguments lately. He asks me if that's not enough to trust him. I tell him we can trust each other but not totally, so he still waits. "* (Participant #3)

Finally, FSWs also mentioned their own intention to use HIVST as a negociation power to encourage their boyfriends to get tested in return for unprotected sex:

*" I told him that if he gets tested, we're going to have unprotected intercourses. But until he does, I won't accept an intercourse without a condom. So when I brought the self-test to him, he accepted it and used it. "* (Participant #3)

*" […] My boyfriend did the self-test when I suggested it to him, otherwise he won't have sex with me anymore. It’s a matter of health or what? Because he's a man; I can't say he's having sex with me alone. […] If he doesn't want to take the test with me, so there's no more [unprotected] sex with me. "* (Participant #11)

### Beliefs about consequences

FSWs’ apprehensions about the possibility of getting an HIV diagnosis were explored. One participant who had obtained a reactive self-test was particularly concerned that she would not be able to marry or have a child. Another was worried that her parents would find out that she was a sex worker. One FSW was worried about dying from the disease. Finally, two participants mentioned the risk of suicide following a reactive self-test result:

*" You know not everyone is brave? There are some who may think about killing themselves, because they will believe that it is all over for them because of a positive result. "* (Participant #22)

*“ In the immediate future, when someone does the self-test alone in his room and finds out that he is sick, he can kill himself, if it is someone who cannot bear to be sick and who does not understand that there is a cure or a painkiller for it. He may seek to harm himself or his reaction may cause him trouble. "* (Participant #25)

### Emotions

FSWs’ emotional response to HIVST was explored in our study. During the individual in-depth interviews, some FSWs said that they were quite serene and calm when they used the HIV self-test:

*" I am used to take care of myself. I was not afraid doing the self-test. But if by any chance my result was positive, I would understand that these are the hazards of the trade. "* (Participant #8)

FSWs’ peace of mind seemed to be linked to their low perception of their personal risk of getting HIV. Many participants felt protected from HIV due to their regular condom use and their good screening habits:

*" I wasn’t afraid, because I hadn't done anything that could bring me this danger. I regularly wear condoms with clients, so there is no reason to be afraid of getting infected with HIV. "* (Participant #20)

*“ I had no fear because I am used to go regularly for a doctor's visit and get tested and I was confident in myself. "* (Participant #27)

Seven women (24.1%), on the contrary, said that they had felt fear or anxiety when using HIV self-tests, or that they had seen such emotions in secondary beneficiaries:

*“ When I did the self-test, my heart pounded when I was waiting for the result; I was overcome with great fear. I was so scared that I sweated profusely. […] My fear was related to the possibility of a result indicating that I am infected; but not to the use of the self-test. "* (Participant #26)

Finally, three participants had previously been tested positive for HIV during standard HIV testing in a health facility, but had denied the diagnosis and refused ART. HIVST enabled these women to see their results for themselves, accept their diagnosis and start a treatment.

*“ Yes, I was told I had HIV, but I didn't believe it. […] As they said that the OraQuick kit is the HIV test, I thought okay I'll check what they said with the OraQuick, if it's true or false. What I did and found yes, that's it when I saw the two lines. "* (Participant #1)

*" [When I took the test last year] the result was positive […] I couldn't believe it. […] So I was sick regularly, then I went to the hospital and the doctor told me it was because of this HIV disease. That's why I did the self-test again and decided to take the treatment. "* (Participant #4)

*“ Since my screening in 2011 which revealed to me my status as an HIV-infected person, I had never been screened again until the beginning of December 2020 when I used the HIV self-test kit to confirm my HIV status. "* (Participant #28)

## Discussion

This theory-based qualitative study aimed to assess the acceptability and feasibility of HIVST for FSWs in Cotonou, Benin, in order to inform the development of a tailored program for Beninese key populations. Eight major domains of the TDF were used to explore the potential determinants of HIVST use: knowledge, reinforcement, social influences, beliefs about capabilities, intentions, behavioural regulation, beliefs about consequences and emotions.

The results indicate that there is a high level of HIVST acceptability within the FSWs population in Cotonou and its surroundings. All the study participants showed interest in using HIVST if made available in Benin. This finding is compatible with the results of several studies conducted in different contexts which show high acceptability of HIVST in key populations [1, 14, 16, 23–35]. HIVST seemed to be particularly attracting for the participants because it promotes autonomy and privacy. When saliva-based, it also is a painless test that is easily accessible. Concerning the different HIVST distribution modalities, the majority of our participants said that they preferred to receive them from NGOs dedicated to FSWs or health care facilities that they already visit on a regular basis. FSWs have also shown great interest in the HIVST secondary distribution. Participants made it clear that the devices have to remain free of charge to maintain their attractiveness to FSWs.

Only two participants (6.9%) had already heard about HIVST before the study. The fact that participants had few or no knowledge about HIVST offered the opportunity to collect answers free from external influences. FSWs low level of knowledge was expected since HIVST is a relatively new screening method that is not yet available in the country. Our participants’ low awareness of HIVST is also consistent with results from other studies conducted in low and middle income countries [1, 29]. Since HIVST is not well known among FSWs and the Beninese general population, it is necessary to raise awareness about it through information campaigns. HIVST national implementation will only be feasible in Benin if mass education programs are first put in place to inform the population about its use and its limitations.

All 29 FSWs interviewed thought HIV self-tests were easy to use without assistance. All participants were confident in their ability to use the device properly and to follow the instructions. However, unlike other research, our study methodology did not allow direct assessment of FSWs’ capacity to use HIVST since there was no observer or evaluator. Still, it is well known that strict instructions adherence is essential to obtain reliable results from HIVST. In fact, many studies have shown that mistakes are frequently made when performing HIVST, especially among populations with low levels of education [29, 36–44]. The interviewer asked participants by which steps they had used the device. While most women were able to describe the key steps, some inconsistencies in their responses were noted, which suggest that errors in execution and interpretation could have been observed if the study methodology had provided this type of direct evaluation. We believe that appropriate, clear and concise user instructions are essential to minimize errors and maximize HIVST performance. In this regard, the majority of participants mentioned that the in-person demonstrations, the manufacturer's illustrated instructions and the advices given by community animators, PEs and health professionals had helped to build their confidence in their ability to use the device properly.

This study highlighted the fact that FSWs experience stigmatization and social exclusion in Benin. In this context, HIVST implementation must be done carefully, to avoid targeting only FSWs. Broadening HIVST access to all high-risk individuals might be a way to mitigate the stigma surrounding HIVST.

HIVST implementation often raises concerns about risk compensation. In our study, although the majority of FSWs said that they did not intend to change their sexual behaviors because of HIVST, some of them tended to stop using condoms with their boyfriends when they obtained non-reactive self-test results. In our opinion, seroselection could be acceptable in a regular couple; however, in an open couple as for FSWs, this strategy requires a high screening frequency from both stable partners. Thereby, HIVST implementation will have to be part of a global prevention program which includes counselling, screening for sexually transmitted infections (STIs) and promotion of consistent condom use. Health care professionals and community animators will have to frequently remind HIVST users that this method does not screen for other STIs and that condom use is still important.

Our study also shows that some clients may use HIVST as a negociation tool to obtain unprotected transactional sex with FSWs. In our opinion, if a client offers a higher price for unprotected sex, the FSW mitigates her risks by first requiring that the client uses the HIV self-test and demonstrates his HIV-negative status. In this situation, if the client agrees to perform the test and obtains a non-reactive result, the FSW has a minimal risk of acquiring HIV by having unprotected sex with this client, especially when HIV incidence is low in the general population. In this context, HIVST serosorting may be a protective practice for FSW, although less effective than consistent condom use. In other words, HIVST seroselection is still safer than unprotected sex with a partner of undetermined HIV status. However, this approach is opposed to WHO official recommendations, which advises to avoid using HIVST for serosorting purposes or to justify high-risk sexual behavior [12].

Three participants in this study used HIVST to retest, after being tested positive through standard HIV testing in a formal health facility in the past. These FSWs had initially denied their diagnosis and refused ART. HIVST enabled them to acknowledge their results, accept their diagnosis and initiate appropriate treatment. This finding is consistent with other research which show that retesting for HIV is frequent among people living with HIV [28, 45, 46]. This result suggests that HIVST could enable people who had previously disengaged to re-engage in care.

In our study, FSWs were encouraged to share HIV self-tests within their social network. This distribution strategy had already been shown successfull in studies conducted in different countries [14, 47–50]. In our study, HIVST secondary distribution was well received among FSWs’ boyfriends and regular clients. In addition, the majority of secondary users asked for FSWs’ help or direct assistance to perform the test. Only three participants reported their boyfriends’ refusal to use the self-tests.

The principal strength of this study is the use of the TDF to categorize and understand factors associated with HIVST acceptability within the FSWs population of Cotonou and its surroundings. Even if it is difficult to explain how the different domains influence each other, the TDF is appropriate to explore a wide range of barriers and enablers to HIVST use. Unlike other studies that used only quantitative methods to explore HIVST acceptability, this study led to a better understanding of participants’ perceptions by using qualitative methods. These qualitative results will help understand the findings from the quantitative part of the study that is in progress.

Another strength of our study is the fact that we capitalized on structures already established in Benin to facilitate our pilot project. We relied on the participation of two user-friendly health centers already dedicated to FSWs and four local NGOs to promote and distribute HIV self-tests. These organizations were in place long before our project, and were already well-known by FSWs. In our opinion, using structures already in place allows us to draw better conclusions about this program feasibility.

The study has a few limitations. Since all the 14 domains of the TDF were not explored, some elements related to HIVST acceptance could have been omitted. However, the TDF was developed in the aim of choosing the most relevant domains according to the situation. Secondly, as for all qualitative studies using interview techniques, our study was subject to social desirability bias; participants may have overestimated their regular condom use or failed to disclose their HIV-positive status. Finally, since the majority of participants (75.9%) has accessed HIVST through community-based distribution, we were not able to gain a good understanding of the experience of FSWs who had been offered HIV self-tests in health facilities (13.8%) or as part of the secondary distribution (10.3%).

## Conclusion

Our study shows a very high level of acceptability for HIVST among FSWs in Cotonou and its surroundings. Results also demonstrate the feasibility of implementing HIV self-tests distribution in Benin. HIVST should be implemented in Benin quickly and free of charge for all individuals at risk of HIV. HIVST offer should be integrated with comprehensive sexual health and prevention services that promote safe sex practices, consistent condom use and regular STI screening.

## Supplementary Information

**Additional file 1.**

## Data Availability

Data sharing is not applicable to this article as no quantitative datasets were generated or analyzed during the current study. The data included in this study are qualitative and sensitive in nature, potentially compromising participant confidentiality. It is possible to obtain an anonymized data set from the corresponding author on reasonable request.

## Abbreviations

AIDS: Acquired immunodeficiency syndrome
ART: Antiretroviral therapy
DIST: Dispensaire IST
FSW: Female sex worker
HIV: Human immunodeficiency virus
HIVST: Human immunodeficiency virus self-testing
NGO: Non-Governmental Organization
PE: Peer educators
STI: Sexually transmitted infections
TDF: Theoretical Domains Framework
UNAIDS: Joint United Nations Programme on HIV/AIDS
WHO: World Health Organisation
