# Supplementary material for: HIV self-testing implementation, distribution and use among female sex workers in Cotonou, Benin: a qualitative evaluation of acceptability and feasibility
Source: BMC Public Health. 2022 Mar 26;22:589. doi: 10.1186/s12889-022-12917-3 (PMC8962183; doi:10.1186/s12889-022-12917-3)
Supplement: Supplementary file 1 — Additional file 1. [file 12889_2022_12917_MOESM1_ESM.docx]

**HIV self-testing implementation, distribution and use among female sex workers in Cotonou, Benin: A qualitative evaluation of acceptability and feasibility**

**Interview guide for female sex workers**

**Introduction**

First, I want to thank you for responding to our invitation and taking the time for this interview. My name is ______. I am a research assistant for the ongoing research project that takes place in Cotonou and its surroundings. This project, as you have certainly heard of, is an interventional research that aims to demonstrate the implementation of the distribution and use of oral HIV self-tests among female sex workers in Cotonou and its surroundings.

The *OraQuick HIV Self-Test* can allow anyone to know their HIV status by taking their own saliva sample, performing the test, and reading the result, often in a private setting, alone or with a trusted person.

Since you have personally received HIV self-tests as part of the project, we have invited you to this interview to gain a better understanding of your experience using the self-tests, but also sharing it among your social networks. The answers you provide will be anonymous. The information collected will be kept confidential. We seek your consent to record the interview to ensure that we collect and fully transcribe your responses without distortion. The results of this research will help improve HIV self-testing promotion programs by adapting them to the needs and contexts.

Note: The comments in brackets are notes for the interviewer and are not directly mentioned to the participants.

**Section 1: Characteristics and profile of the participant, her environment, and social networks**

1. Can you tell me about yourself and your sex work activities? Can you tell me about your social networks (working and living environments)?

- How old are you?
- For how long have you been practicing sex work? On which sites do you practice?
- What do you do other than sex work to earn money?
- What is your nationality?
- What is your religion?
- What is the highest level of education you have reached (primary, secondary, or higher)?
- What is your marital status (married, divorced, separated, widowed)?
- Do you have a boyfriend or a regular sexual partner?
- Do you currently live with your husband, boyfriend, or regular sexual partner?
- How many biological children do you have?
- At this moment, how many dependents (children, parents, or others) are in your care?

**Section 2: Experience with HIV self-tests use**

2. Have you ever been tested for HIV in the past? How so (in a health center, or by NGO facilitators), and on what occasion?

3. How many times have you been screened for HIV in your life? How often do you get tested for HIV? When was the last time you were screened for HIV?

4. Did you know about HIV self-tests before receiving them as part of the project?

- How did you hear of HIV self-testing?
- According to your understanding, how do these self-tests work?

5. Tell me about the HIV self-tests you received.

- How many did you receive?
- Where did you receive them?
- Who did you receive them from (health professional, NGO educator, colleague)?
- What did you do with it?
- How many self-tests have you used for yourself?
- If you have not used all the self-tests you received, why?
- What did you do with the self-tests that you did not use for yourself?

6. What did you think of your experience using the HIV self-test?

- Did you have any concerns about the HIV self-test result? Would you say HIV self-testing is stressful?
- Since the community agents distribute self-tests as part of the project, would you say that they are easily accessible?
- Before or during the self-tests distribution, did you follow a demonstration on its use? If yes, who offered the demonstration? Did you receive instructions for its use? Were these explanations clear?
- Have you seen pictures, watched a video or listened to audio messages to learn how to use HIV self-tests?
- Were these images, videos, and audio messages clear?
- Did you receive general advice on what to do once you get the self-test result? Did you know what to do if your result was positive (go to a testing center for confirmation and management)? Did you know what to do if your result was negative (repeat the test regularly, every three months, due to continued exposure to the risk of infection)?
- From the moment you received the HIV self-test, how long did it take you to use it?
- Where did you use your self-test? Why did you choose this place?
- Under what circumstances did you use your self-test (alone, or with the assistance of a trusted person)? Why did you choose to do it this way?
- What difficulties did you face when using the self-test? Were the instructions difficult to follow? Do you think you made any mistakes in performing the test? Did you need help to use the test? If yes, who helped you?
- Which steps were the most complicated or difficult with the self-test (collecting the saliva sample, handling the device, reading and interpreting the results)?
- Was it difficult to read the self-test result?
- Do you feel like you are able to use an HIV self-test alone?
- Did you know your serological status following the use of the HIV self-test?

7. What are the main benefits that you see with HIV self-testing, after having experienced it, compared to the conventional screening services at the health center? What does this new screening method bring to you?

8. What are the main limitations or disadvantages that you see with HIV self-testing, after having experienced it, compared to conventional screening services at the health center?

**Section 3: Experience with HIV self-tests secondary distribution**

9. Did you recommend HIV self-tests use to others? Why?

9a. Were you used to talk about sexually transmitted infections screening with these people?

9b. How many HIV self-tests have you shared with others?

9c. Can you tell me about each self-test you have shared?

- Who did you share self-test with (boyfriend, regular client, occasional client, friend, FSW colleague, relative)?
- Why did you choose to share self-tests with these people?
- Tell me how the sharing process went.
- What strategies did you use to introduce the self-test to these people?
- Where and when did this happen?
- How did you feel offering the self-test (was it easy, difficult, awkward, etc.)?
- What was their reaction when you offered them the HIV self-test?
- Were there any refusal or reluctance to use the self-test? Why?
- What strategies did you use to convince the person to use the HIV self-test?
- Do you know if the people you shared self-tests with actually used them?
- Did these people prefer to use the self-test alone or with your assistance?
- Did these people share the self-test result with you?
- Have you told the person what to do when he/she gets a positive or negative result?
- How did people react when they learned their results?

10. Can you tell me about any change in your sexual behaviors following the experience of using and/or sharing self-tests?

- Has there been any changes in sexual behaviors between you and your sexual partners after you used the self-test?
- Has there been any changes in condom use (consistent use VS discontinuation of condom use) with your regular or occasional intimate partners? If so, what explains this change?
- What do you think is the impact of the use of self-tests on sexual behavior?

11. Tell me about what you enjoyed most in this HIV self-test sharing experience.

- What advantages and benefits do you see?
- Would you say you have been a role model by sharing and demonstrating self-tests use to members of your social networks? Were you proud to play this role?

12. Tell me about the things or situations that you did not like about this HIV self-test sharing experience.

**Section 4: Negative or harmful events, social prejudices**

13. Tell me about any negative or harmful event that you might have experienced or witnessed in connection with HIV self-tests distribution, use or sharing.

- Have you been victim of coercion, violence (physical, verbal, sexual, attempted suicide), discrimination, or stigmatization in connection with self-tests distribution and use?
- Have you been victim of violence (physical, verbal or sexual) by an intimate partner resulting from self-tests use?
- Have you witnessed cases of coercion related to self-tests distribution and use?
- Have you heard rumors of possible cases of coercion or violence in connection with self-tests distribution and use?
- Have you sold or purchased self-tests? Have you received any solicitations to buy or to sell self-tests?
- Have you heard any rumor or witness the sale of self-tests that were distributed as part of the study?

14. Have you experienced situations where you felt stigmatized in connection with HIV self-testing?

- Were you worried that someone might know you were in possession of an HIV self-test?
- Has the fear of being stigmatized because of an HIV diagnosis discouraged you from using a self-test?
- How do you think people around you (boyfriend, family, friends) would react if they knew you had an HIV self-test in your possession?
- In your opinion, what do FSWs think of HIV self-testing in general?
- Would you be comfortable getting an HIV self-test in a public place (e.g., pharmacy)?

**Section 5: For women who spontaneously report having had a positive result with the self-test, experience of linkage to care and treatment services**

15. After realizing that your self-test was positive, did you go to a health center? Why did you go there? Were you able to get a confirmation test?

16. How were you linked or referred to care services? Was it complicated? What were the barriers to access to care?

**Section 6: Suggestions and recommendations to improve HIV self-tests distribution and use**

17. Should HIV self-tests be distributed in other places that you think are easier to access? Through which channel would you like to receive HIV self-tests?

18. How many self-tests would you like to receive during an upcoming distribution session? Were three self-tests enough?

19. What could be done to improve your personal experience of self-tests use?

20. Were the information materials and instructions provided clear and helpful? How could they be improved?

21. In your opinion, what type of help or assistance is best to carry out HIV self-testing (direct assistance from a health worker at the health center, assistance from a community worker, telephone call from a community worker, assistance from a FSW colleague, view of the demonstration video, listening to the audio messages)?

**Section 7: Suggestions and recommendations to improve HIV self-tests secondary distribution**

22. What could be done to improve the process of HIV self-tests secondary distribution to sexual partners and other members of your social networks?

Do you have questions?

Thank you for participating in this interview.
